# Epidemiological Study on Sand Flies in an Endemic Focus of Cutaneous Leishmaniasis, Bushehr City, Southwestern Iran

**DOI:** 10.3389/fpubh.2015.00014

**Published:** 2015-02-02

**Authors:** Mohammad Darvishi, Mohammad Reza Yaghoobi-Ershadi, Farideh Shahbazi, Amir Ahmad Akhavan, Reza Jafari, Hassan Soleimani, Nastaran Yaghoobi-Ershadi, Mohammad Khajeian, Hossein Darabi, Mohammad Hossein Arandian

**Affiliations:** ^1^School of Public Health, Tehran University of Medical Sciences, Tehran, Iran; ^2^Esfahan Health Research Station, National Institute of Health Research, Esfahan, Iran; ^3^Yazd Health Research Station, National Institute of Health Research, Yazd, Iran; ^4^Polytechnic University of Madrid, Madrid, Spain; ^5^Deputy of Health Services, Bushehr University of Medical Sciences, Bushehr, Iran; ^6^The Persian Gulf Tropical Medicine Research Center, Bushehr University of Medical Sciences, Bushehr, Iran

**Keywords:** epidemiology, Iranian sand flies, *Phlebotomus sergenti*, *Phlebotomus papatasi*, *Leishmania turanica*, *Leishmania major*

## Abstract

Cutaneous leishmaniasis is the most important health problem in the city of Bushehr, southwestern Iran. The objective of the study was to determine some ecological aspects of sand flies in the city during 2010–2011. Sand flies were collected monthly from outdoors and indoors by sticky traps at four selected districts of the city. They were also dissected and examined by nested-PCR for identification of the parasite during August–September of 2011. A total of 1234 adult sand flies were collected and 6 species including 3 of Genus *Phlebotomus* and 3 of Genus *Sergentomyia* were identified. Four species including *P. papatasi* (3.98%), *P. sergenti* (1.14%), *S. tiberiadis* (87.18%), and *S. baghdadis* (7.7%) were found indoors. Six species including *P. papatasi* (3.47%), *P. sergenti* (3.17%), *P. alexandri* (0.1%), *S. tiberiadis* (77.74%), *S. baghdadis* (15.41%), and one female of *S. clydei* (0.11%) were collected from outdoors. Sand flies started to appear from March and disappear at the end of January. There was only one peak in the density curve in July. The study revealed that *S. tiberiadis* and *S. baghdadis* could enter indoors which 89 and 81.8% of them were found blood-fed, respectively. Moreover, *P. papatasi*, *S. tiberiadis*, and *S. baghdadis* were active indoors and outdoors in most months of the year. Nested-PCR of *P. papatasi* females was positive against kinetoplast DNA of *L. major* and *L. turanica* and also mixed natural infections were found by *L. gerbilli* and *L. turanica*. Moreover, mixed infections by *L. major* and *L. turanica* were observed in this species. *Sergentomyia clydei* and *S. tiberiadis* were found to be negative to any DNA of *Leishmania* species. *Phlebotomus sergenti* females were found infected with DNA of *L. turanica* and this is the first report of natural infection and detection of the parasite from this sand fly species in worldwide.

## Introduction

There is a long history of Cutaneous Leishmaniasis (CL) in Iran. The oldest traditional medical book has been written by an Iranian scientist, Avicenna (IbnSina, born in 980, died in 1037), which was completed in 1025, about 1000 years ago. It is called Qanun (The Laws of Medicine) and it was used as a textbook until eighteenth century in the universities of European and Islamic countries. In this book, Avicenna has mentioned on cutaneous lesions of his patients, which was called Khyroonieh, with long duration and the treatment of the ulcers had been difficult and resistant to different drugs, the clinical signs of the ulcers were imagined to be CL (1). The impact of the disease on human health in this part of middle-east was not really recognized until 1940s, since then Iranian leishmaniasis has been the subject of an epidemiological program directed by Ansari, Hadjian, Mofidi, Pooya, Mesghali, and Nadim (2), constitutes an increasing public health problem in the country.

Cutaneous leishmaniasis is endemic in two forms in Iran, Anthroponotic Cutaneous Leishmaniasis (ACL) and Zoonotic Cutaneous Leishmaniasis (ZCL). About 20,000 cases of leishmaniasis are reported annually, which 80% of them are ZCL, 0.5% Visceral Leishmaniasis (VL), and the rest is ACL. *Phlebotomine* sand flies of Iran have been studied since 1930 by a limited number of Iranian and foreign entomologists such as Adler, Theodor, and Lourie but Mesghali was the first Iranian to conduct basic studies on sand flies in this country (3).

Cutaneous leishmaniasis has been epidemic during the years 1988, 1997, and 2008 in the city of Bushehr (Health center of Bushehr province, unpublished data). The causative agents of the disease are *Leishmania major* and *Leishmania tropica*. In some parts of the city, *Tatera indica* is the main reservoir host and *Nesokia indica* as the secondary reservoir. The prevalence of scar was 5.9% among the inhabitants and for ulcer it was <0.5% in 2010 (4). Bushehr is one of the most important free trade industrial zones of the country and the Bushehr Nuclear Power Plant, which is unique in terms of its technology in the Middle East is located 12 km, southeast of the city along the Persian Gulf so lots of people travel around and some make several trips in a year for business.

Bushehr, like most of other Iranian cities, has expanded quickly over recent years. Mass emigration to the city from other parts of the province and urbanization of peripheral with poor facilities and sanitation, construction of buildings nearby rodent colonies, increase of non-endemic people in south Pars Project, Bushehr Military Complex, and the presence of Bushehr Nuclear Power Plant are the main reasons of occurrence of CL in the city. If the disease does not receive considerable attention by the health authorities, it may spread into other parts of the country, which are free from CL. However, the entomological studies on sand flies have not been carried out in the city yet and there is no accurate data on vector(s) of the disease.

The objective of this study was to determine some ecological aspects of sand flies in the city of Bushehr during 2010–2011, as an initial step in the development of effective strategies for the control of leishmaniasis in the city.

## Materials and Methods

### Study area

The city of Bushehr located in a plain running along the coastal region on the Persian Gulf coast of southwestern Iran and is the administrative center of its province.

**Figure 1:**
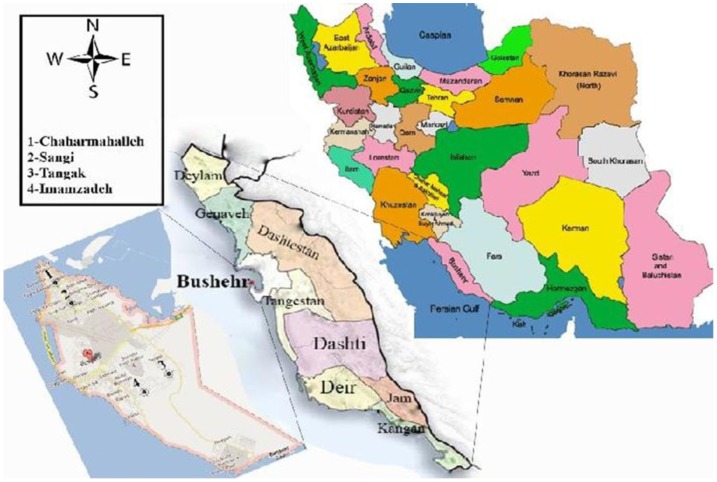
**Map of the city of Bushehr, showing the geographical location and study sites**.

Field studies were carried out during 2010–2011, in the city of Bushehr (Latitude: 28°55′ 30″ N, Longitude 50°50′ 17″ E, altitude: 5 m above sea level) (Figure 1). The city had a population of 221,016 in 2011, while this was 133,753 in 1991 with an increase about twofolds in the last two decades. The area has a hot desert climate though it does receive more rainfall than most cities on the Persian Gulf. The rain is confined to the period from November to May, when temperature is pleasantly mild and is extremely erratic. The long summer from April to October is brutally hot, humid, and completely rainless. In 2010, the maximum and minimum mean monthly temperature was 39 and 12.1°C in August and February, respectively, and the total annual rainfall was 4.29 mm with a minimum of 0.1 mm in May and 2.45 mm in February. The minimum mean monthly relative humidity was 58% (December) and the maximum was 74% in January (Bushehr Meteorological Organization, unpublished data).

### Sand fly sampling and monitoring

To obtain enough data, four infected districts of the city were selected, called Chaharmahalleh in the north, Sangi in the center, Tangak in the south, and Imamzadeh in the southwest. Sand flies were collected monthly from fixed places of indoors (bedrooms, sitting rooms, toilets, bathrooms, store rooms, hallways) and outdoors (rodent burrows, base of walls, and cracks in it in the yards) fixed places, using 30 sticky traps (castor oil coated white papers 20 cm × 35 cm) from the beginning (March) to the end of active season (January). Collected sand flies were stored in 70% ethanol. For species identification, sand flies were mounted in Puri’s medium, produced at the medical entomology department (5), and identified after 24 h using the keys of Theodor and Mesghali (6). In case of molecular studies, sticky traps were used to collect sand flies at the end of transmission season from indoors and outdoors in September 2011, stored in 96% ethanol at −20°C until examination and all fed and gravid females were tested individually by nested-PCR for identification of *Leishmania* parasite.

### DNA extraction

The middle parts of female sand flies (including thorax and abdomen) were used; the samples were washed with absolute ethanol and after drying washed three times in cold sterile phosphate-buffered saline (PBS; pH 7.2). Before submitting the sandflies to the DNA extraction procedure, they were subjected to 13 freeze/thaw cycles, using liquid nitrogen and boiling water and sampler tips or pestle, to disrupt tissue and treated as described for the tissue samples (4). Genomic DNA was extracted and purified using Qiagen extraction Kit (Qiagen, 69504) according to the manufacturer’s manual with the minor modification of increasing incubation time to 5 min to increase the yield of DNA in the final step. DNA was stored at −20°C until analysis. The concentration of extracted DNA was measured spectrophotometrically by NanoDrop (Thermo Fisher Scientific, USA).

### Molecular assays

#### Primer design for amplification of ITS2

Primers designed previously and used to amplify a 230 bp product in *L. major*, a 206 bp in *L. gerbili*, and a 141 bp in *L. turanica* across the internal transcribed spacer 2 [Akhavan et al. (7)]. The external primers, Leish out F (5’-AAA CTC CTC TCT GGT GCT TGC-3’) and Leish out R (5’-AAA CAA AGG TTG TCG GGG G-3’), and internal primers, Leish in F (5’-AAT TCA ACT TCG CGT TGG CC-3’), and Leish in R (5’-CCT CTC TTT TTT CTC TGT GC-3’) were selected to distinguish among the parasite species in a nested-PCR system (7).

#### Nested-PCR

We used nested-PCR to identify the *Leishmania* species. Conditions and parameters for PCR were as previously described with the minor modification (8). All samples were tested in 25 μl amplification reaction mixtures with 12.5 μl of the master mix (Taq DNA polymerase, 2× Master Mix Red, Amplicon, Germany), 1.8 μl of primers, 10.7 μl H_2_O, and 1 μl of template DNA. The first-round PCR was performed based on the following conditions: initial denaturation at 95°C for 5 min; followed by 35 cycles including denaturation at 95°C for 30 s, annealing at 56°C for 30 s, and extension at 72°C for 45 s; and a final extension at 72°C for 5 min. The second-round (nested) PCR was performed as the same first-round exception for annealing at 58°C for 30 s. At the end, 10 μl of the reaction mix was analyzed by 2.5% agarose gel electrophoresis.

Additionally, for all PCR reactions, one negative control without DNA and one positive control with standard DNA were included to confirm the results of two rounds of nested-PCR. The PCR products of the negative and positive controls of the first-round PCR were used as negative and positive controls in the second round, respectively. Finally, 10 μl of the PCR products were loaded on 2.5% (W/V) agarose gels, and stained with ethidium bromide to visualize by electrophoresis. Initially, ITS-PCR was confirmed with standard DNA of reference strains *L. major* (MRHO/IR/75/ER), *L. gerbilli* (MRHO/CN/60/GERBILLI), and *L. turanica* (MRHO/SU/1983/MARZ-051) as positive controls and distilled water were used as negative controls (7, 8).

#### PCR-RFLP analysis

PCR products (20 μl) were digested with *Mnl*I 2 μl at 37°C for 4 h without prior purification using conditions recommended by the supplier (Fermentas Life Sciences, Germany). The restriction fragments were subjected to electrophoresis in 3% agarose gel containing ethidium bromide for 3 h at 65 V and visualized on a UV transilluminator.

## Results

### Sand fly species

**Figure 2:**
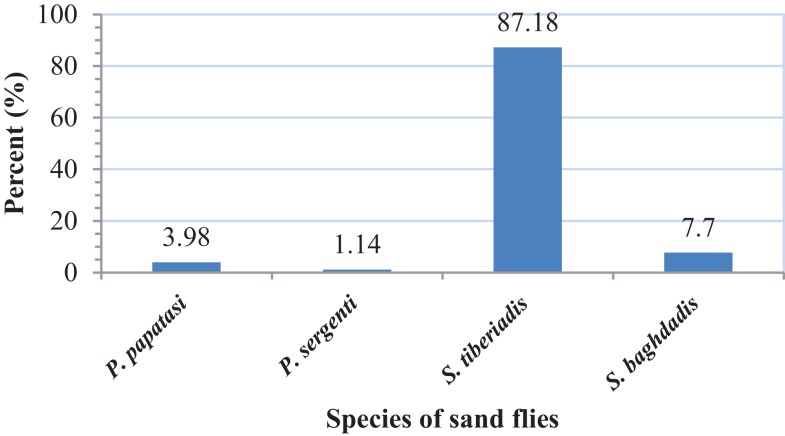
**Fauna and percent of collected sand flies from indoors, Bushehr city, Iran**.

**Figure 3:**
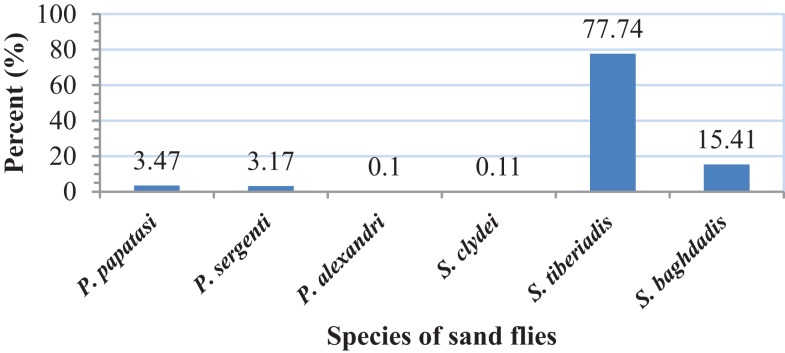
**Fauna and percent of collected sand flies from outdoors, Bushehr city, Iran**.

A total of 1234 adult sand flies, 882 from outdoors and 352 from indoor resting places were collected and identified. The following four species were found indoors: *P. papatasi* (3.98%), *P. sergenti* (1.14%), *S. tiberiadis* (87.18%), and *S. baghdadis* (7.7%). From outdoors, six species including *P. papatasi* (3.47%), *P. sergenti* (3.17%), *P. alexandri* (0.1%), *S. tiberiadis* (77.74%)*, S. baghdadis* (15.41%), and *S. clydei* (0.11%) were collected (Figures 2 and 3). The study revealed that *S. tiberiadis* and *S. baghdadis* could enter indoors, which 89 and 81.8% of them were found blood-fed, respectively.

**Figure 4:**
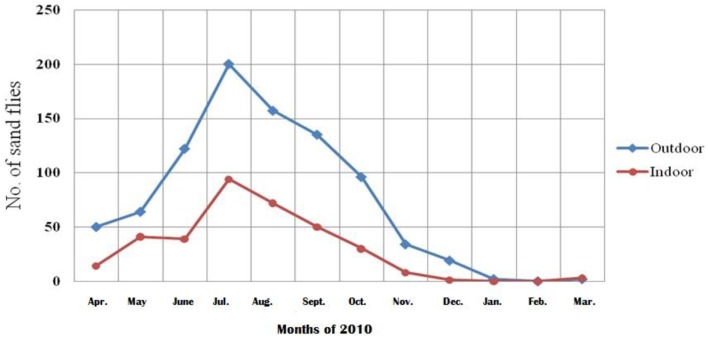
**Monthly prevalence of sand flies in the city of Bushehr, Iran, 2010–2011**.

The sand flies started to appear in April and disappeared at the end of January. There was only one peak in the density curve in July (Figure 4). Moreover *P. papatasi, S. tiberiadis*, and *S. baghdadis* were active indoors and outdoors in most months of the year. Sand flies were active 10 months in the city and the decrease of sand fly density at the end of January was most probably due to the rains. No sand fly was found in the city during February and March due to cold weather.

### *Leishmania* infection of sand flies

**Figure 5:**
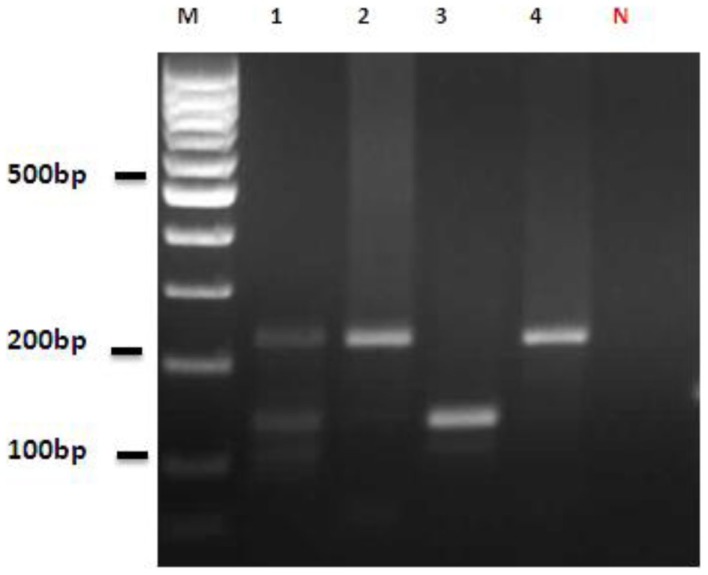
**Nested-PCR amplification of DNA extracted from infected sand flies and reference strains**. Lane M, 100 bp DNA ladder (Fermentas); Lane 1, mixed infection of *Leishmania gerbilli* and *Leishmania turanica* detected from *Phlebotomus papatasi*; Lanes 2 and 4, reference strains, *Leishmania gerbilli*; Lane 3: reference strain, *Leishmania turanica*; Lane N, negative control (distilled water).

**Figure 6:**
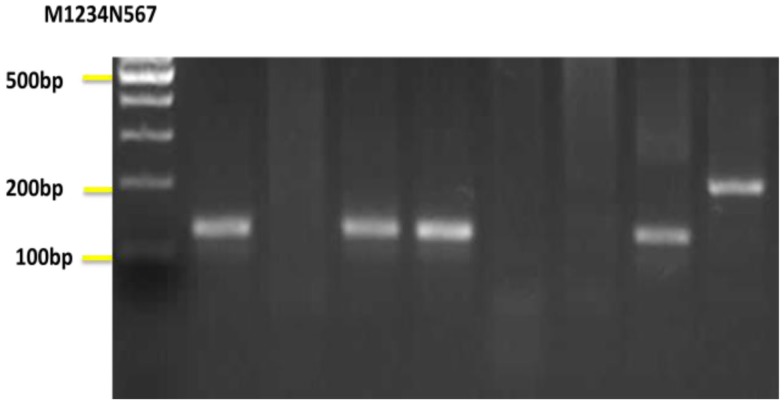
**Nested-PCR amplification of DNA extracted from sand flies**. Lane M, 100 bp DNA ladder; Lanes 1,4,6 *Leishmania turanica* detected from *Phlebotomus papatasi*; Lane 3, *Leishmania turanica* detected from *Phlebotomus sergenti*; Lane 7, *Leishmania major* detected from *Phlebotomus papatasi*; Lane N, negative control; Lanes 2,5 negative samples.

**Figure 7:**
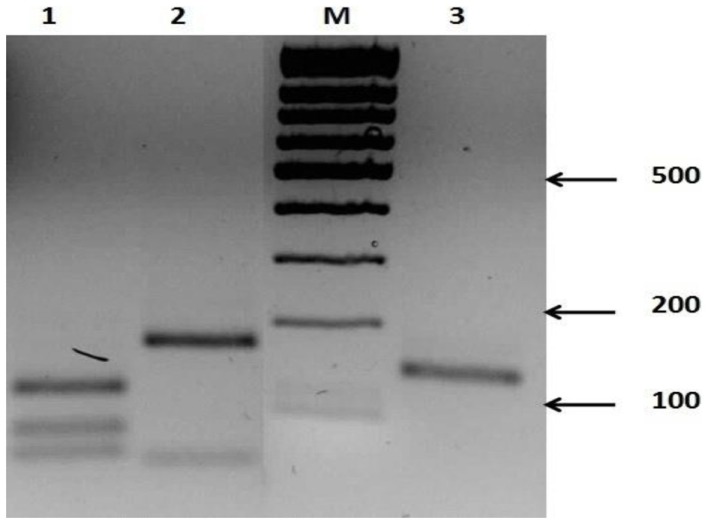
**Restriction products of nested-PCR amplicons in three species of *Leishmania* after digestion with Mnl1**. Lane M, 100 bp DNA ladder; Lane 1, *Leishmania major*; Lane 2, *Leishmania gerbilli*; Lane 3, *Leishmania turanica*.

Twenty individual female specimens including four species of *P. papatasi*, *P. sergenti*, *S. tiberiadis*, and *S. clydei* were tested against *Leishmania* parasite DNA. *Leishmania* DNA was found in 11 (55%) out of 20 specimens. Table 1 shows the natural *Leishmania* infection rate of sand flies by nested-PCR and Figures 5–7 show the patterns of ITS-PCR for sand flies. One out of four female *P. papatasi* from outdoors and four of six from indoors were found to be infected by *L. major* and two of four from outdoors *by L. turanica*, which produced species-specific bands of 231 and 141 bp, respectively. Mixed natural infections with *L. gerbilli* and *L. turanica* were also observed in 16.7% of *P. papatasi* from indoors. In rodent burrows, mixed infections of both *L. major* and *L. turanica* was found in one of two of this sand fly species.

**Table 1:** **Natural *Leishmania* infection rate (%) of sand flies by nested-PCR in the city of Bushehr, Iran, September 2011**.

Capture site	Species	No. of examined	Leishmania infection rate (%)			
			*L. major*	*L. turanica*	*L. gerbilli*	*L. major*
					+	+
					*L. turanica*	*L. turanica*
Outdoors	*P. papatasi*	4	25 (1/4)	50 (2/4)	–	–
	*P. sergenti*	3	–	66.6 (2/3)	–	–
Indoors	*P. papatasi*	6	–	66.7 (4/6)	16.7 (1/6)	–
	*P. sergenti*	2	–	–	–	–
	*S. tiberiadis*	1	–	–	–	–
Rodent burrows (outdoors)	*P. papatasi*	2	–	–	–	50 (1/2)
	*S. tiberiadis*	1	–	–	–	–
	*S. clydei*	1	–	–	–	–

*Figures in parentheses are numbers of positive/no. of examined specimens and figures out of parentheses are percent of positive*.

Two out of three female *P. segenti* from outdoors were found to be infected by *L. turanica* and produced a PCR band of 141 bp. One of the infected *P. sergenti* was gravid and the other was semi-gravid and both of them were collected by sticky traps near dwellings.

## Discussion

In the present study, three *Phlebotomus* and two *Sergentomyia* species were identified for the first time in the city of Bushehr. *Phlebotomus papatasi*, *P. sergenti*, and *P. alexandri* have medical importance because of their proven or probable roles as vectors of parasites causing human leishmaniasis in the Old world (9). *Phlebotomus papatasi* and *P. sergenti* are known to feed readily on humans (3, 10).

Anthroponotic cutaneous leishmaniasis caused by *Leishmania tropica* represents a serious medical problem in several countries in the Middle-East region, including Iran. *Phlebotomus sergenti* is one of the proven vectors of *L. tropica* in some of these countries (11–14). This species is considered to be the probable vector of ACL in 14 endemic foci located in eight provinces of Iran (3). In the present study, *P. sergenti* represented 3.17 and 1.1% of all *Phlebotomus* caught from outdoors and indoors, respectively. It was active 4 months (April, May, July, August) in indoors and 7 months in outdoors and has been caught from all infected districts except Sangi district located in the center of the city. *Phlebotomus sergenti* is rarely found indoors and its density is very low as 4 male specimens of this species were caught but 28 of these flies were collected from outdoors during August–October in the city. Whether we could not find natural infection of females of this sand fly species by *L. tropica*, it has been recorded from the cities of Esfahan in the center and Shiraz in the south by molecular and monoclonal diagnostic antibody tests (3, 15). *Phlebotomus sergenti* has a wide distribution in the country and extends beyond the distribution of *L. tropica*.

*Phlebotomus alexandri* is widely distributed in Palaearctic region, but it is never common (6). It is usually considered as a mountain species (16–18) although it occurs in some low land areas as well (17). It is thermophilic and moderately hydrophilic species and aggressive to human beings (19). The present results indicate that this species is a new record in this coastal area of Bushehr, Iran. It is suspected vector of *L. infantum* and *L. major* in the provinces of Fars and Khuzestan, southern Iran (3).

*Sergentomyia tiberiadis* is a thermophilic and xerophilos species, usually in low and dry rocky mountains of southern Afghanistan, but absent in humid areas. In Iran, it is found in eight provinces, five in the south and the others in the center, northwest, and northeast. Regarding its restricted distribution to Pakistan, Afghanistan, and Iran and specially its presence in human residences in the areas of CL, vectoral role of this species needs to be investigated. *Sergentomyia baghdadis*is distributed in Iraq, Iran, Pakistan, and southern Afghanistan, it is thermophilic and hydrophilic species of plains, sometimes numerous in human dwellings and rodent burrows. Whether it is considered as a possible vector of reptilian leishmaniasis, its feeding habits and relation to CL should be investigated as well.

In the current study, we found DNA of *L. major* and *L. turanica* and mixed infection *of L. gerbilli* and *L. turanica* in *P. papatasi*, which is agreement with the findings by Strelkova et al. in 1996 and Parvizi and Ready in 2008 (20, 21) indicate the possible transmission of both *L. major* and *L. turanica* by *P. papatasi*. This sand fly species is considered as the vector of *L. major* to humans in the city of Bushehr. *Sergentomyia clydei* and *S. tiberiadis* were found to be negative for *Leishmania*.

*Phlebotomus sergenti* was found naturally infected by *L. turanica* near dwellings and according to our knowledge this is the first report of infection of this sand fly species by *L. turanica* in worldwide. Natural infection of *P*. *sergenti* was found in an experimental study by Chajbullinova and her colleagues in 2012; they showed that in *P. sergenti*, *L. turanica* promastigotes were present only on the defecation of blood meal remnants (22). In a study in China by Li-Ren and colleagues in 1995, *P. mongolensis* and *P. anderjevi* have been found naturally infected in the field and also in experimental studies (23). In Turkmenistan and Uzbekistan, Strelkova and her colleagues detected *L. turanica* from *P. papatasi, P. anderjevi, P. caucasicus, P. mongolensis, P. alexandri*, and *S. clydei* in natural foci of ZCL (20). In Iran, *L. turanica* has also been detected from *P. papatasi* in central and north of the country and also from *P. caucasicus* in the northwest (3). Reports of *L. turanica* from other *Paraphlebotomus* species suggest that vector competence for *Leishmania* may differ between members of this subgenus. However, further studies are needed to clarify the role of these vectors in the circulation of *L. turanica*. This *Leishmania* species has also been detected repeatedly from *R. opimus* in Iran, Uzbekistan, China, Kazakhstan, and Mongolia. It has also been detected from *T. indica* and *Rattus norvegicus* in the city of Bushehr and also from *Nesokia indica* in southwest of the country (4).

In 2014, two isolates from two patients in northeast of Iran were examined by Nested-PCR-RFLP and sequenced several times. They were identified as *L. turanica* haplotype TurkHo3, which was previously isolated and identified from sand flies and rodents in this region (24). It can be approved as causative agents of ZCL by more extensive sampling and followed by standardized molecular diagnosis. Specific entomological and epidemiological studies including monitoring annual fluctuations of *P. papatasi* and *P. sergenti*, different aspects on sand fly ecology in the infected districts of the city, drawing attention to the diagnosis and treatment of *Leishmania* infections, follow up studies on more of *Leishmania* species from *P. sergenti* in the area are necessary in order to reach a better understanding of the interaction between *L. major* and *L. turanica* are recommended. The management of organic waste, controlled urbanization, and improvement of sanitary condition in the suburbs would reduce to a significant degree the density of sand fly vectors.

## Conflict of Interest Statement

The authors declare that the research was conducted in the absence of any commercial or financial relationships that could be construed as a potential conflict of interest.
